# Rifampicin-Mediated Metabolic Changes in *Mycobacterium tuberculosis*

**DOI:** 10.3390/metabo12060493

**Published:** 2022-05-29

**Authors:** Soujanya D. Yelamanchi, Archita Mishra, Santosh Kumar Behra, Gayathree Karthikkeyan, Thottethodi Subrahmanya Keshava Prasad, Avadhesha Surolia

**Affiliations:** 1 Molecular Biophysics Unit, Indian Institute of Science, Bangalore 560012, India; soujanyay@iisc.ac.in; 2 Telethon Kids Institute, Perth 6009, Australia; archita.mishra@telethonkids.org.au; 3 Center for Systems Biology and Molecular Medicine, Yenepoya Research Center, Yenepoya University, Mangalore 575018, India; santoshb@yenepoya.edu.in (S.K.B.); gayathreekarthikk@gmail.com (G.K.); keshav@yenepoya.edu.in (T.S.K.P.)

**Keywords:** bacteria, RNA polymerase inhibitor, global metabolomics, targeted metabolomics, ABSciex QTRAP 6500 mass spectrometer

## Abstract

*Mycobacterium tuberculosis (Mtb)* is considered to be a devastating pathogen worldwide, affecting millions of people globally. Several drugs targeting distinct pathways are utilized for the treatment of tuberculosis. Despite the monumental efforts being directed at the discovery of drugs for *Mtb*, the pathogen has also developed mechanisms to evade the drug action and host processes. Rifampicin was an early anti-tuberculosis drug, and is still being used as the first line of treatment. This study was carried out in order to characterize the in-depth rifampicin-mediated metabolic changes in *Mtb,* facilitating a better understanding of the physiological processes based on the metabolic pathways and predicted protein interactors associated with the dysregulated metabolome. Although there are various metabolomic studies that have been carried out on rifampicin mutants, this is the first study that reports a large number of significantly altered metabolites in wild type *Mtb* upon rifampicin treatment. In this study, a total of 173 metabolites, associated with pyrimidine, purine, arginine, phenylalanine, tyrosine, and tryptophan metabolic pathways, were significantly altered by rifampicin. The predicted host protein interactors of the rifampicin-dysregulated *Mtb* metabolome were implicated in transcription, inflammation, apoptosis, proteolysis, and DNA replication. Further, tricarboxylic acidcycle metabolites, arginine, and phosphoenolpyruvate were validated by multiple-reaction monitoring. This study provides a comprehensive list of altered metabolites that serves as a basis for understanding the rifampicin-mediated metabolic changes, and associated functional processes, in *Mtb,* which holds therapeutic potential for the treatment of *Mtb*.

## 1. Introduction

*Mycobacterium tuberculosis (Mtb)* predominantly infects the lungs of immunocompromised individuals, causing tuberculosis (TB) [1]. According to the 2021 World Health Organization (WHO) report, approximately 9.87 million people were infected with TB globally, of which 56% were men, 33% were women, and 11% were children. Moreover, 1.5 million deaths were reported worldwide with 15% of TB fatality ratio. Global estimates have shown that TB is considered to be the second leading cause of death due to a single pathogenic agent. Treatment of resistant strains—multidrug-resistant (MDR) and extremely drug-resistant (XDR) TB—has become a major concern, as the success rate for treating MDR/RR-TB is 59%, and for XDR-TB is 52% [2].

Rifampicin (RIF), a front-line of anti-tubercular agent, has been used in the treatment of TB for over half a century [3]. It is known to inhibit RNA polymerase by interacting with the β subunit of the enzyme, thus inhibiting the process of transcription [4]. It inhibits RNA synthesis by blocking the elongation step, thereby preventing protein synthesis and leading to bacterial killing [5,6]. There is a significant number of growing shreds of evidence on the acquisition of mutations in the *Mtb* β subunit of the RNA polymerase gene *rpoB* that lead to pathogenic resistance against RIF [7]. Further, drug tolerance to RIF is also observed in *Mtb* through mechanisms involving the enhanced activity of efflux pumps, metabolic shifts, and overexpression and mistranslation of the *rpoB* gene [8,9,10]. Despite the existence of drug tolerance and the emergence of resistance mechanisms in *Mtb*, the use of RIF in combination with other drugs is cataloged in the WHO model list of essential medicines 2021 [11]. Moreover, the TB treatment regimen includes RIF in the treatment of latent TB infections under *Mtb* drug susceptible conditions, as per the guidelines mentioned by the National Tuberculosis Controllers Association (NTCA) and Centers for Disease Control (CDC) and Prevention 2020 [12].

A couple of metabolomic studies have been reported in the recent past on RIF mutant strains of *Mtb*. A study has previously reported 16 differential metabolites by GC–MS analysis in two RIF mutant strains of *Mtb* [13]. In another study, GC–MS analysis resulted in the identification of 15 metabolites in wild type and S531L and S522L RIF mutant strains of *Mtb* [14]. Similarly, 87 metabolite features in methanol extraction, and 99 features in chloroform fraction, have been reported to be significant in two *rpoB* mutant strains, compared to wild-type *Mtb* [15]. In addition to these studies, metabolomic analysis has also been carried out on RIF-treated *Mycobacterial* strains. Man D.K et al. have reported a number of differential metabolites in RIF-treated *M. smegmatis* [16]. Further, LC–MS analysis has shown 122 altered metabolites in *Mtb* cells treated with RIF at 0.1 to 6.4 µg/mL for a period of 24 h to 72 h [9]. These metabolites were majorly classified as carboxylic acids, amino acids, and tricarboxylic acids and their derivatives. They also included purines nucleosides, pyrimidines, primary aliphatic amines, and carbohydrates and its conjugates.

Metabolites, the reactants of the metabolic pathways, are the vital signaling molecules in the cell. These represent the phenotype of the cell by governing various physiological functions through metabolite–protein interactions [17,18]. Over the past decade, metabolomics has advanced rapidly in areas of biomarker discovery, drug development, and precision medicine [19]. As RIF continues to be the first-line drug for the treatment of TB, it is essential to understand the global metabolic perturbations caused by this drug in *Mtb.* In this study, we performed a metabolomic analysis of *Mtb* upon RIF treatment, and, unlike previous studies, here we were able to identify 157 non-redundant metabolites that have not been previously reported. We performed global and targeted metabolomic analyses at the MS2 level using a high-throughput mass spectrometer to identify the dysregulated metabolites by RIF in *Mtb*.

## 2. Results

### 2.1. MS2 Identification of Mtb Metabolites by RIF Treatment

**Figure 1:**
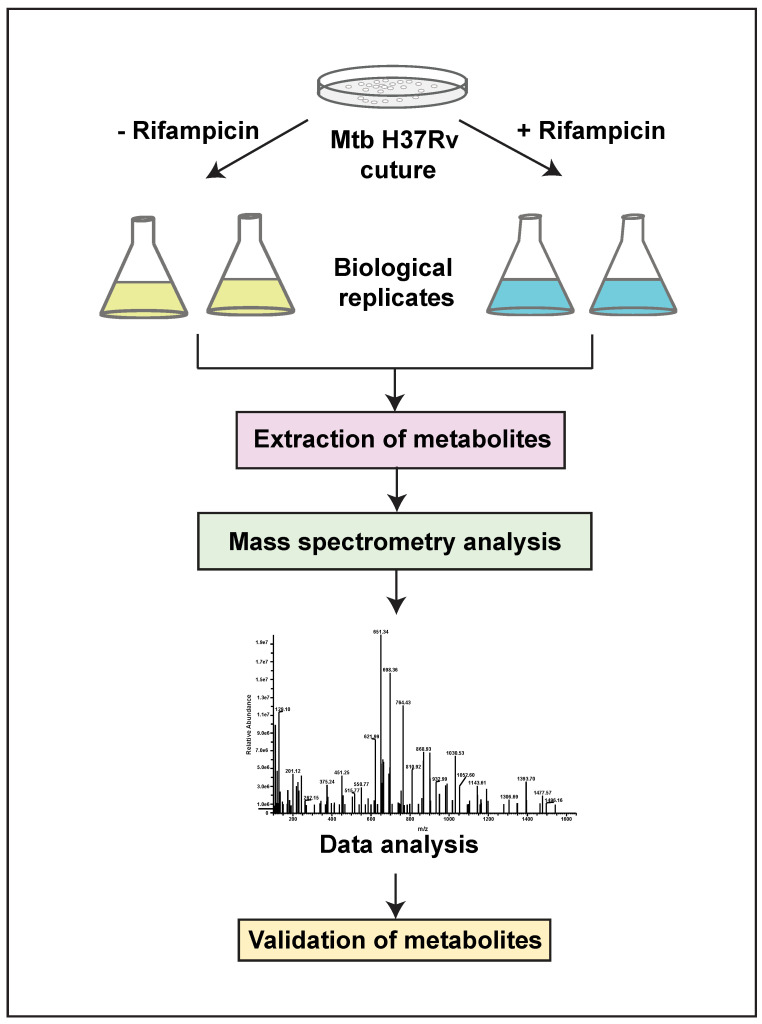
A schematic illustration of the metabolomics pipeline, with details of sample conditions and experimental workflow.

**Figure 2:**
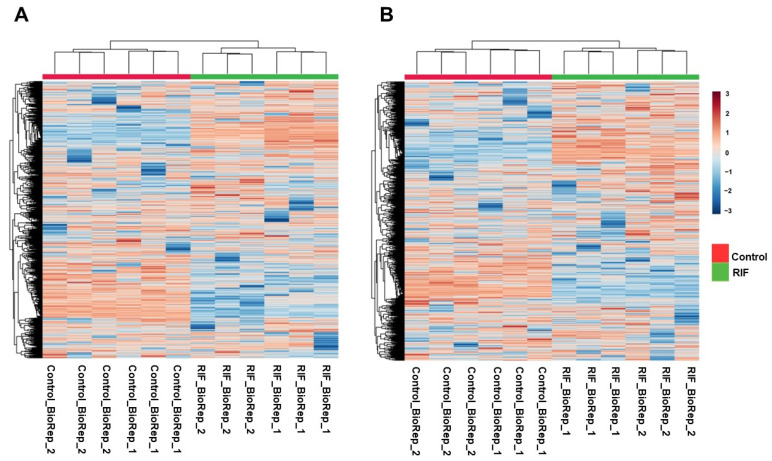
Hierarchical clustering heat map showing metabolic features identified in untreated and RIF-treated *Mtb* samples in: (**A**) Positive mode (**B**) Negative mode.

Mass spectrometry analysis of *Mtb* lysates in the presence and absence of RIF treatment was carried out in triplicate runs for each biological replicate. A schematic illustration of the experimental and analysis pipeline is shown in Figure 1. LC–MS/MS analysis led to the identification of 2290 aligned peaks in positive mode and 2311 aligned peaks in negative mode. The list of aligned peak features in positive and negative modes is tabulated in Supplementary Materials, Tables S1 and S2. Of these, 2224 aligned peak features in positive mode and 2258 aligned peak features in negative mode were observed in two technical replicates for each biological group. Collectively, 461 features in positive mode and 280 features in negative mode were mapped, at the MS2 level, to the *Mtb* H37Rv strain of KEGG [20] and BioCyc [21] databases, for the first time, in this study. Hierarchical clustering of *Mtb* and RIF-treated aligned peak features showed significant clustering of replicates and discrete separation between the groups. This analysis was carried out separately for data acquired from positive and negative modes, as shown in Figure 2A,B.

The mass spectrometry-derived data quality was examined by comparing control and RIF-treated sample runs with the intermediate blank runs to rule out the possibility of sample carry over [22]. Hence, PCA analysis was carried out, which showed discrete separation of sample groups from blank runs. Further, PLS discriminant analysis (PLS-DA) of sample groups and blank runs showed variable importance in projection (VIP) scores of important features that were either insignificant or undetected in the blank runs [23]. The schematic illustrations of PCA and PLS-DA analyses are provided in Supplementary Materials, Figure S1.

### 2.2. Metabolic Dysregulation in Mtb by RIF

**Figure 3:**
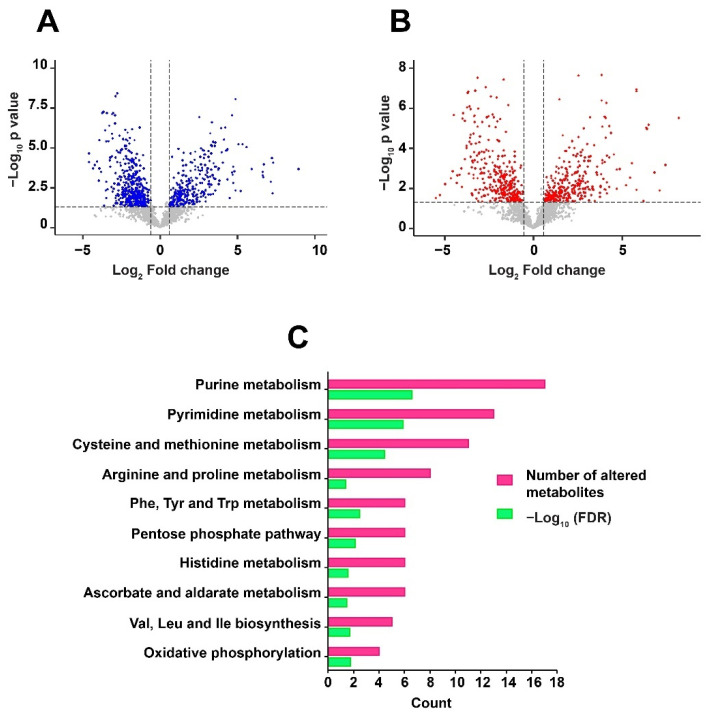
Volcano plots show the distribution of significantly altered metabolites in: (**A**) Positive mode and (**B**) Negative mode. The dotted line on *X*-axis represents p-value cut off while the dotted line on *Y*-axis shows the fold change cut off. (**C**) Metabolic pathways enriched with False Discovery Rate (FDR) ≤ 0.05 against the dysregulated metabolites are shown as a horizontal multi-set bar graph. The length of bars represents the count for both FDR and the number of metabolite entities enriched per pathway.

Differentially regulated metabolites were analyzed using an unpaired *t*-test to calculate the *p*-values. Metabolites with a fold change of 1.5 and *p*-value ≤ 0.05 were considered to be significantly altered. A significant dysregulation of 680 metabolite features in positive mode, and 631 metabolite features in negative mode, was observed in response to RIF in *Mtb*. Of these, 101 metabolites in positive mode and 75 metabolites in negative mode were non-redundantly assigned to *Mtb* databases. Drugs, alkaloids, and other mapped exogenous compounds were deleted for downstream analysis [24]. Differential expression of the identified metabolites, including significant and non-significant, are depicted as volcano plots in Figure 3A,B. A partial list of differentially expressed metabolites, with significant raw *p*-values (≤0.05), is given in Table 1. The entire list of significantly altered metabolites, in both positive mode and negative mode, is provided in Supplementary Materials, Tables S3 and S4, respectively.

### 2.3. Metabolic Pathway Analysis and Classification of Metabolites

Metabolic pathway analysis plays a significant role in understanding the physiological processes associated with the altered metabolites. Pathway enrichment was carried out against *Mtb* H37Rv using MBROLE [25]. KEGG identifiers were used as input to perform the analysis. About 10 pathways including purine metabolism, pyrimidine metabolism, phenylalanine, tyrosine and tryptophan biosynthesis, arginine and proline metabolism were enriched with significant False Discovery Rate (FDR) ≤ 0.05. The pathways associated with dysregulated metabolites are shown in Figure 3C. Global metabolomics led to the identification of metabolites classified as nucleotides-deoxyribonucleotides and ribonucleotides; nucleosides, pyrimidines, amino acids-glutamine, threonine, proline and arginine; water-soluble vitamins—riboflavin and biotin; fatty acids and carbohydrates.

### 2.4. Prediction of Host Protein Targets against RIF Mediated Mtb Dysregulated Metabolites

**Figure 4:**
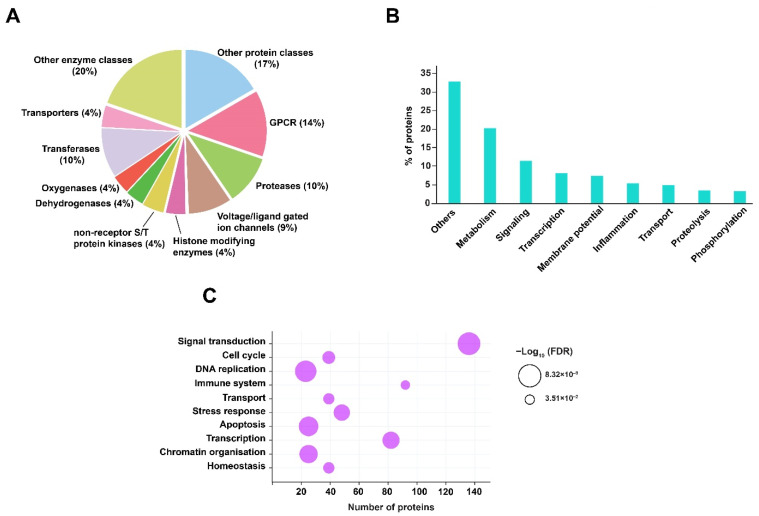
GO and pathway analysis of predicted protein interactors. (**A**) Classification of protein targets is represented as a pie chart. (**B**) Biological processes are shown as a vertical bar graph. (**C**) Pathway analysis is represented as a bubble plot. The size of the bubble signifies the FDR value (≤0.05).

Prediction of host protein targets provides a better understanding of the host biological or functional processes that are regulated in response to RIF treatment and *Mtb* infection. Henceforth, in this study human protein targets were predicted against the dysregulated metabolites, using the tool BindingDB. BindingDB comprises a large number of protein and small molecule interaction datasets, essentially derived from experimental studies [26]. In order to identify the protein targets, SMILES identifiers of the dysregulated metabolites were used as inputs for the tool. A total of 351 human interactors were obtained with a similarity score ≥85% from BindingDB (Supplementary Materials, Table S5). Gene ontology (GO) analysis of these protein targets was performed by acquiring GO terms from the PANTHER database. The majority of the protein targets were classified as G-protein coupled receptors, proteases, transferases, and voltage- or ligand-gated ion channels. In addition to these, protein classes, such as histone modifying enzymes, non-receptor serine/threonine protein kinase, dehydrogenases, oxygenases, and other enzymes and protein classes, were associated with the protein targets. These proteins are majorly involved in signaling, metabolism, membrane potential, transcription, and inflammatory processes. Other biological processes impacted include protein phosphorylation, proteolysis, and transport. The GO analysis, including protein classes and biological processes of the predicted protein targets, is shown in Figure 4A,B.

The predicted proteins were subjected to pathway analysis using the Reactome pathway database. Pathways such as signal transduction, immune system, cellular response to stress, cell cycle, homeostasis, DNA replication, and programmed cell death were identified with significant FDR ≤ 0.05. The pathway enrichment of predicted protein targets in response to RIF and *Mtb* infection is shown in Figure 4C.

### 2.5. Multiple Reaction Monitoring (MRM)-Based Validation of Altered Metabolites

**Figure 5:**
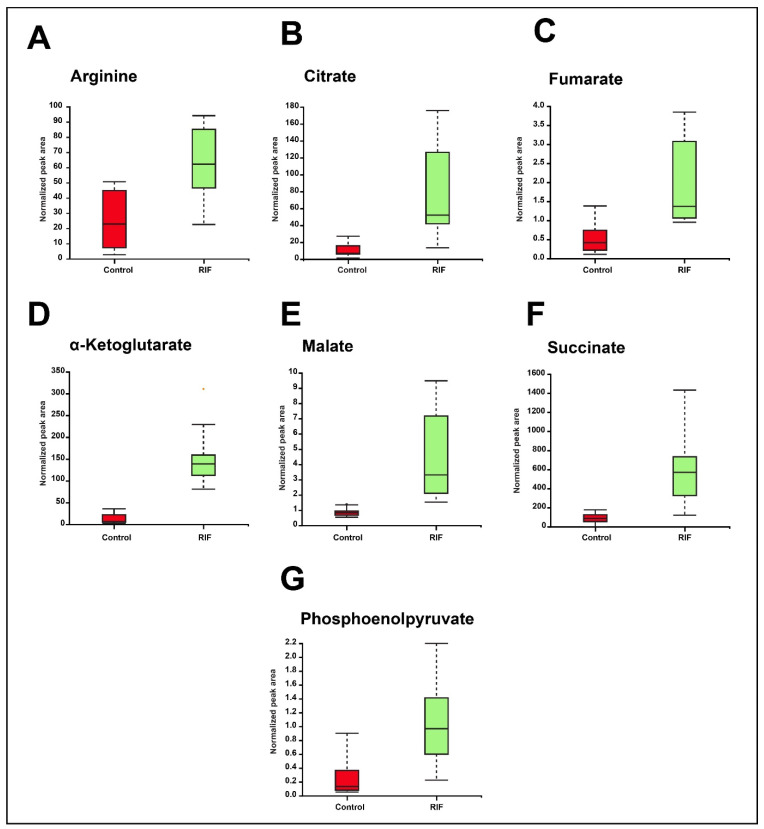
Box-whisker plots showing significant dysregulation of MRM validated metabolites in RIF and untreated or control groups. (**A**) Arginine. (**B**) Citrate. (**C**) Fumarate. (**D**) α-Ketoglutarate (**E**) Malate. (**F**) Succinate. (**G**) Phosphoenolpyruvate.

In this study, 14 metabolites belonging to the tricarboxylic acid (TCA) cycle, the arginine metabolism, and certain amino acids were validated by using a mass spectrometry-based Multiple Reaction Monitoring (MRM) approach. A total of seven metabolites, including α-ketoglutarate, citrate, malate, fumarate, succinate, phosphoenolpyruvate, and L-arginine were dysregulated with significant *p* value (≤0.05). The fold change values and *p*-values, along with the optimization and transition details of the validated metabolites, are shown in Supplementary Materials, Table S6. Box-whisker plots of the significantly dysregulated metabolites are shown in Figure 5.

## 3. Discussion

Although there was a previously published study on RIF-treated *Mtb*, 157 metabolites were uniquely identified in this study compared to a previously published study [9]. Unlike the previous study, 461 features in positive mode and 280 features in negative mode were mapped to the *Mtb* database at the MS2 level, which represents a comprehensive number of metabolites identified in this study for the first time. Moreover, both studies significantly differ in terms of drug concentration, duration of treatment, and culture growth phases. In this study, the MABA assay showed inhibition of *Mtb* growth with RIF treatment at 5 ng/mL to 320 ng/mL concentration with an IC_50_ value of 10.17 ng/mL and MIC_90_ of 40 ng/mL (Supplementary Materials, Figure S2). Hence, *Mtb* cells were treated with RIF at a concentration of 40 ng/mL, which was used to study the metabolic changes in *Mtb*. RIF is known to mediate *Mtb* killing through hydroxy radical formation within the pathogen [27]. As expected, monodehydroascorbate radicals (2.4-fold, ≤0.05 *p*-value) and isonicotinoyl radicals (7.5-fold, ≤0.05 *p*-value) were upregulated by RIF treatment in *Mtb*. In contrast, anti-oxidants, such as ergothioneine and mycothiol-bimane conjugate, an intermediate of mycothiol biosynthesis, were downregulated in this study. Ergothioneine and mycothiol, as low molecular weight antioxidants, confer protection to *Mtb* against oxidative species [28,29,30].

The TCA cycle is vital for the maintenance of cell homeostasis, as the intermediates of the TCA cycle are involved in the synthesis of proteins, fatty acids, and nucleotides, in addition to distinct signaling mechanisms [31]. Metabolites of the TCA cycle—citrate, malate, α-ketoglutarate, succinate, and fumarate, validated by LC-MS/MS-MRM approach—were found to be upregulated in this study. Glutamine, the connecting link between the TCA cycle, purine, and pyrimidine metabolism, was also upregulated by RIF. Purines such as GTP, XTP, dATP, and dADP, and pyrimidines, including CDP, dCDP, dCTP, and dTMP, were downregulated by RIF. In contrast ADP, cAMP, UTP, UDP, dUMP, TDP nucleotides were upregulated by RIF. RIF exposure led to dysregulation of the balance of nucleotide synthesis and mRNA degradation, whereby cytosine, guanosine, thymine, and thymidine were also found to be altered [14].

Previous reports have shown aggregation of cholesterol on the cell wall of mycobacteria, which is known to decrease its permeability to the standard of care anti-TB drugs, such as RIF. In addition to cell permeability, cholesterol accumulation on the cell wall shields the recognition of mycobacterial antigens, as previously reported with a 20% decrease in antibody binding, thus protecting the bacteria from host immunity. Mutational studies have shown that the 3-ketosteroid Δ^1^-dehydrogenase (KstD) enzyme, which converts androst-4-ene-3,17-dione (AD) to androsta-1,4-diene-3,17-dione (ADD), is an essential step for cholesterol utilization or degradation in *Mtb*. Further, the growth of H37Rv and the kstD mutant was inhibited when grown in cholesterol-depleted media [32]. FadD3, an 3aα-H-4α(3′-propanoate)-7aβ-methylhexahydro-1,5-indanedione (HIP)-CoA synthetase, converts HIP to HIP-CoA in the cholesterol degradation pathway. A *Rhodococcus jostii* FadD3 mutant showed reduced growth when compared to a FadD3 mutant complemented with *Mtb* enzyme in 1 mM of cholesterol-supplemented media [33]. ADD and HIP, associated with the cholesterol degradation pathway, were upregulated by RIF.

Menaquinones play a key role in the electron transport chain that transfers electrons to terminal oxidases and reductases, mediating ATP synthesis [34]. Decreased expression of menaquinone-9 in *Mtb* has been shown to result in reduced oxygen consumption, ATP production, and bacterial survival in the presence of inhibitors. Previous reports have shown that inhibitors targeting menaquinone-9 biosynthetic enzymes—MenA and MenG—serve as potential anti-bacterial agents in controlling *Mtb* growth [35,36]. Menaquinone synthesis begins with chorismite, the product of the shikimate pathway [37]. In addition to menaquinone-9, aromatic amino acids such as phenylalanine, tyrosine, and tryptophan are also synthesized through the downstream reactions of chorismite [38]. In this study, menaquinone-9 and chorismite are downregulated by RIF. These dysregulated metabolites also correlate with perturbation of metabolites related to the electron transport chain; NADH, ADP, diphosphoric acid, and triphosphoric acid were upregulated, while ATP was downregulated, in this study.

MEP pathway metabolites, including D-Glyceraldehyde 3-phosphate, 2C-Methyl-D-erythritol 2,4-cyclodiphosphate, 4-CDP-2-C-methyl-D-erythritol 2-phosphate, and farnesyl diphosphate, were found to be upregulated in *Mtb* in response to RIF. Mutational studies have shown that the *idsA* gene, involved in the synthesis of geranyl pyrophosphate from farnesyl pyrophosphate, is vital for the growth of *Mtb* [39].

Prediction of host protein targets against *Mtb* infection and RIF treatment resulted in the observation of interesting host biological processes. Previous reports indicate that RIF prevents the augmented expression of apoptosis-associated proteins in *Mtb* infected macrophages. Moreover, previous experiments have shown RIF-mediated inhibition of apoptosis and nitic oxide production [40]. Further, cytokine levels, including IL-10, IFNγ, and TNFα, have been reported to decrease by isoniazid and RIF in *Mtb* infected macrophages [41].

In this study, global and targeted analyses were performed on data acquired at the MS2 level. MRM validation was performed on some of the key metabolites associated with the TCA cycle and arginine metabolism. In the last couple of years, mass spectrometry-based metabolomics has evolved in the employment of quality control methods, which have progressed from the comparison of blank profiles to the currently deployed pooled QC samples [22]. Although the deployment of quality-control sample runs, along with the main sample runs in the mass spectrometer, has become a standard practice, this concept of QC was introduced at the time the data were acquired for this study [42]. Hence, sample runs were compared to the blank runs to avoid carryover of sample features (see Supplementary Materials, Table S1). Here, high confidence data were acquired at the MS2 level for both global and targeted metabolomic approaches, and most of the altered metabolites identified in this study provide insights for understanding the role of RIF in *Mtb*.

## 4. Materials and Methods

### 4.1. Bacterial Culture

*Mtb* H37Rv cells (kind gift from Dr. Amit Singh, IISc, Bangalore) were grown up to logarithmic phase (0.6 OD) in 100 mL Middlebrook 7H9 media containing 0.05% Tween 80 (Sigma, Burlington, MA, USA) and 10% OADC. Successively, actively growing *Mtb* cells were treated with 40 ng/mL RIF (Sigma, Burlington, MA, USA) in 0.5% DMSO (Sigma, Burlington, MA, USA), while the non-drug-treated cells were treated with 0.5% of DMSO as a control. The cultures were incubated at 37 °C for 12 h, and the cell density was adjusted to 0.4 OD (~0.6 × 10^8^ cells) following cell harvest. The cultures and treatment were carried out in biological duplicates.

### 4.2. Bacterial Cell Lysis and Metabolite Separation

Control and RIF-treated *Mtb* cultures were centrifuged at 5000× *g* rpm at 4 °C for 10 min to separate the pellets. The extracted pellets were washed thrice with ice-cold PBS and were snap-frozen with liquid nitrogen. Instantaneously, the frozen pellets were resuspended in an ice-cold resuspension buffer comprised of methanol, acetonitrile (ACN), and water at a 2:2:1 ratio. Subsequently, cell lysis was carried out by mechanical disruption with zirconia beads (0.1 mm) using a tissue homogenizer. The cell lysates were subjected to centrifugation at high speed for 20 min and the supernatants were collected and filtered through 0.22 µm (Corning, New York, NY, USA) filters. Further, samples of both conditions were vacuum dried before tandem mass spectrometry analysis.

### 4.3. Tandem Mass Spectrometry Analysis for Untargeted Metabolomics

The dried metabolite samples were analyzed on quadrupole ion-trap-ABSciex QTRAP 6500) (SCIEX, Framingham, MA, USA) mass spectrometer in technical triplicates for each biological duplicate sample, in positive mode and negative mode, separately. The instrument was connected in line with the liquid chromatography system (Agilent 1290 Infinity II), which contained a C_18_ column (RRHD Zorbax; 20 × 150 mm, 1.8 μm particle size). A 30 min LC method was set up for the separation of metabolites using solvent A (0.1% formic acid in water) and solvent B (0.1% formic acid, 90% can) at a flow rate of 0.3 mL/min. Solvent B, with a gradient of 2.0% for 1 min, 2.0–30% for 9 min, 30–60% for 7 min, 60–95% for 9 min, and 2% for 4 min, at a flow rate of 0.3 mL/min and sample injection volume of 15 µL, was applied for LC-MS/MS analysis. Data were acquired using the IDA method, comprised of enhanced mass spectra (EMS) and enhanced product ion (EPI) modes, which are inbuilt in the ABSciex QTAP 6500 mass spectrometer. The most intense five MS1 spectra (EMS mode) were subjected to MS/MS analysis (EPI mode) using collisional-induced dissociation (CID). Metabolite raw data were acquired with a probe temperature of 450 °C in both positive (4500 V) and negative polarities (−4500 V). Samples were run with a cycle time of 2.091 sec per cycle. The parameters of collision energy (CE) and de-clustering potential (DP) were set at 45 V and 75 V, respectively. Blank runs were executed on mass spectrometer amidst triplicate sample runs to avoid carryover of metabolite features from adjacent samples.

### 4.4. Data Analysis and Metabolite Mapping

Mass spectrometry-derived data were analyzed on MZmine (Version 2.53) [43], as described previously [44]. The mzML files, derived from wiff files, were uploaded to MZmine to obtain the RT, *m*/*z*, and peak areas of the identified features. Initially, the files including control and RIF-treated were subjected to mass detection at the MS1 and MS2 levels, where the peak intensities were set to a minimum. The precursors, containing fragment details, were selected by the MS/MS peak list builder to build the *m*/*z* feature list. The features that passed 0.05 Da *m*/*z* tolerance through the Peak Extender algorithm were subjected to chromatogram deconvolution, where the noise peak height was set to 1.5 × 10^2^ while the retention time and *m*/*z* tolerance for MS2 pairing were set to 1 min and 0.1 Da, respectively. The deconvoluted features were further processed using an isotopic peaks grouper algorithm, with *m*/*z* and RT tolerance set to 0.25 Da and 0.2 min, respectively. These features were aligned using the Join-Aligner algorithm, with parameters set as follows: *m*/*z* weight, 70%; *m*/*z* tolerance, 50 ppm; RT threshold, 30%; and RT tolerance, 0.5 min. The features were then gap-filled using the Peak finder algorithm, with an intensity tolerance of 30%, RT tolerance of 0.6 min, and *m*/*z* tolerance of 0.05 Da. The resulting duplicate peaks were discarded by employing the New Average mode in the duplicate peaks filter algorithm, with *m*/*z* and RT tolerance set to 0.1 Da and 0.2 min, respectively. The results comprising of peak areas, RT, *m*/*z*, and feature ID were exported at the MS2 level to csv files. Subsequently, precursor masses and their corresponding fragment details were also exported for metabolite assignment. The wiff files of intermediate blank runs in between the sample runs were similarly analyzed, along with the control and RIF-treated sample groups, in MZmine to check for details of sample carry over.

The mgf files derived from MZmine were uploaded to the MS2 Compound tool [45] to attain details of metabolites at the MS2 level. Metabolites specific to Mtb H37Rv were downloaded and annotated from the BioCyc and KEGG databases. The metabolites SMILES ID were submitted to the Competitive Fragmentation Modeling-ID (CFM-ID) tool [46] to retrieve theoretical fragmentation data, which was used to search against experimental MS2 data with precursor tolerance of 0.05 Da and fragment tolerance of 0.5. In addition, at least 2 fragment matches against the theoretical database were set as criteria for metabolite mapping. Further, metabolites with the highest mS-score and rank 1 were chosen to filter the redundant metabolites.

### 4.5. Bioinformatic Analysis

The statistical analysis for this study was performed using MetaboAnalyst online software (Version 5.0; https://www.metaboanalyst.ca/) [47]. Peak areas of the identified features were normalized using median mode to calculate the fold changes. Heat maps were developed using the Pearson distance measure and average clustering method for log_10_ transformed and auto-scaled positive and negative mode data. Pathway analysis and metabolite classification were performed in MBROLE (Version 2.0; http://csbg.cnb.csic.es/mbrole2/) [25] by providing *Mtb* H37Rv as the source organism. Protein classes and biological processes for the predicted protein interactors were analyzed on PANTHER (Version 16.0; http://www.pantherdb.org/), while the pathway analysis for these interactors was performed using REACTOME.

### 4.6. MRM-Based Validation

Targeted analysis was carried out with 14 standards that were individually optimized for RT, DP, EP, CE, and CXP, in addition to Q1 and Q3 masses. In order to optimize MS/MS parameters, around 1 mg of each of the 14 standards were suspended in either 0.1% formic acid or 50% methanol, depending on solubility of the standards. Subsequently, the resuspended standards, at a concentration of 1 ug/mL, were directly infused into the mass spectrometer for optimization of DP, CE, EP, and CXP. Later, the RT and LC gradients were optimized by pooling each standard at a concentration of 1 ug/mL. Mass spectrometry-based MRM analysis was carried out on an ABSciex triple quadrupole mass spectrometer in technical triplicates, per biological duplicate samples. The metabolite extracts from samples were injected into LC-RHP Zorbax C_18_ column (2.1 mm × 150 mm, 2.7 μm; Agilent Technologies, Santa Clara, CA, USA) through an instrument-coupled automated autosampler. Metabolite separation was achieved with a 35 min LC gradient, where the flow rate was set to 0.3 mL/min for solvent A (0.1% formic acid) and solvent B (1% formic acid in 90% ACN), and sample injection volume was set to 15 μL. Solvent B was applied with a gradient of 2% for 3 min, 2–10% for 2 min, 10–30% for 2 min, 30–70% for 7 min, 70–98% for 9 min, and 2% for 7 min. The data were obtained using MRM scan mode in both the polarities. Metabolites were ionized using curtain gas at 20 psi, ion source gas 1 at 25 psi, and ion source gas 2 at 5 psi in ESI. Further, ion spray voltage at 5500 V, collision activated dissociation gas at medium, and ESI source temperature at 450 °C were maintained during the analysis. The data, including *m*/*z*, RT, and peak areas of the standard metabolites, were extracted from Skyline software [48] by uploading the mass spectrometer-derived wiff files. The MRM data, including RT, Q1 and Q3 masses, and the corresponding DP, CE, and CXP values for the validated metabolites, are given in Supplementary Materials, Table S6. In this study, a total of 14 metabolites were relatively quantified in both the modes. The above-mentioned 14 standards were used as analytical standards, while epicatechin was used as an internal standard.

### 4.7. Microplate Alamar Blue Assay (MABA)

An MABA assay was used to determine the MIC_90_ and IC_50_ of RIF. *Mtb* H37Rv cells were grown in sterile 96-well plates up to log phase of 0.6 OD_600_ in Middlebrook 7H9 media supplemented with 10% OADC. A total volume of 200 µL media, containing 10^5^ *Mtb* cells, was dispensed into each well. The wells containing *Mtb* cells without inhibitor served as a positive control, while wells containing only inhibitor served as a negative control, for the experiment. Plain media was used as a blank. The concentration of RIF, ranging from 2.5 ng/mL to 320 ng/mL, was aliquoted into respective wells and incubated for 5 days at 37 °C. Subsequently, 20 µL of Alamar Blue dye was added to each well, in the dark, and re-incubated at 37 °C for 24 h to record the fluorescence intensity. The readings were taken with excitation at 530 nm and emission at 590 nm, and the inhibition percentage was calculated simultaneously. The drug efficacies were represented in terms of IC_50_ and MIC_90_ values. The experiment was performed in triplicates for each of the duplicate samples.

### 4.8. Data Availability

RIF-treated and control *Mtb* metabolomics data were submitted to the publicly accessible MetaboLights database [49]. The MetaboLights database is an archive of metabolite data pertaining to structures, spectra, concentration, biological roles, and localization that were acquired from metabolomic experiments. The metabolite details of this study can be accessed from the database with the identifier MTBLS4243.

## 5. Conclusions

In this study, global and targeted approaches, performed using high-resolution mass spectrometry, led to the identification of a comprehensive number of significantly altered metabolites. In addition to purine and pyrimidine metabolism, the altered metabolites were also associated with oxidative phosphorylation, phenylalanine, tryptophan, and tyrosine metabolism. Moreover, the metabolic pathways and predicted human interactors identified in this study will provide insights for a deeper understanding of the functional processes regulated by RIF in *Mtb*. This is the first study that reports a large number of dysregulated metabolites in *Mtb* by RIF.

## Figures and Tables

**Table 1:** A partial list of altered metabolites.

S.No	Metabolite	Mode of Acquisition	Fold Change	*p*-Value
1	2C-Methyl-D-erythritol 2,4-cyclodiphosphate	Positive	3.33	0.01
2	L-Glutamine	Positive	48.68	0.00
3	Thymidine	Negative	0.47	0.02
4	Thymidine-5′-phosphate	Negative	0.10	0.00
5	Uridine-5′-diphosphate	Positive	5.42	0.01
6	Deoxycytidine diphosphate	Positive	0.09	0.00
7	2-Isopropylmaleic acid	Positive	1.70	0.03
8	3-Deoxy-D-arabino-heptulosonate-7-phosphate	Positive	14.13	0.00
9	4-Guanidinobutyric acid	Positive	5.93	0.02
10	Biotin	Negative	2.14	0.02
11	Cyclic AMP	Negative	9.25	0.00
12	Histidinal	Positive	1.83	0.00
13	L-Cystathionine	Negative	3.95	0.03
14	Menaquinone-9	Negative	0.19	0.00
15	S-Adenosyl-L-homocysteine	Negative	4.09	0.03

## Data Availability

Metabolomics data of this study is available at the MetaboLights database with the study identifier MTBLS4243.

## Supplementary Materials

The following supporting information can be downloaded at: https://www.mdpi.com/article/10.3390/metabo12060493/s1, Figure S1: PCA analysis showing distinct clustering of blank, control and RIF treated samples in (A) Positive and (B) Negative modes. Important features identified by PLS-DA analysis in (C) Positive and (D) Negative modes are depicted; Figure S2: Representative MABA assay to determine MIC90 and IC50 of RIF in Mtb H37Rv cells. (A) 96-well plate showing the change in colour from pink to blue with increasing RIF concentration. (B) Plot showing cell viability against RIF concentration; Table S1: List of metabolite features identified in positive mode; Table S2: List of metabolite features identified in negative mode; Table S3: A complete list of significantly dysregulated metabolic features identified in positive mode; Table S4: A complete list of significantly dysregulated metabolic features identified in negative mode; Table S5: List of predicted human protein interactors of the altered metabolites; Table S6: Details of MRM validated metabolites. The table provides the list of validated metabolites along with FC, *p*-values, transitions and optimization parameters.

## Abbreviations

TB	Tuberculosis
RIF	Rifampicin
PCA	Principal Component Analysis
PLS-DA	PLS discriminant analysis
FDR	False Discovery Rate
GO	Gene Ontology
ACN	Acetonitrile
MRM	Multiple reaction monitoring
TCA	Tricarboxylic acid
FC	Fold change
QC	Quality control
DP	Declustering Potential
RT	Retention time
CE	Collision Energy
EP	Entry Potential
CXP	Cell exit potential
MABA	Microplate Alamar blue assay
