# Supplementary figures and images for: Rifampicin-Mediated Metabolic Changes in Mycobacterium tuberculosis
Source: Metabolites. 2022 May 29;12(6):493. doi: 10.3390/metabo12060493 (PMC9228056; doi:10.3390/metabo12060493)

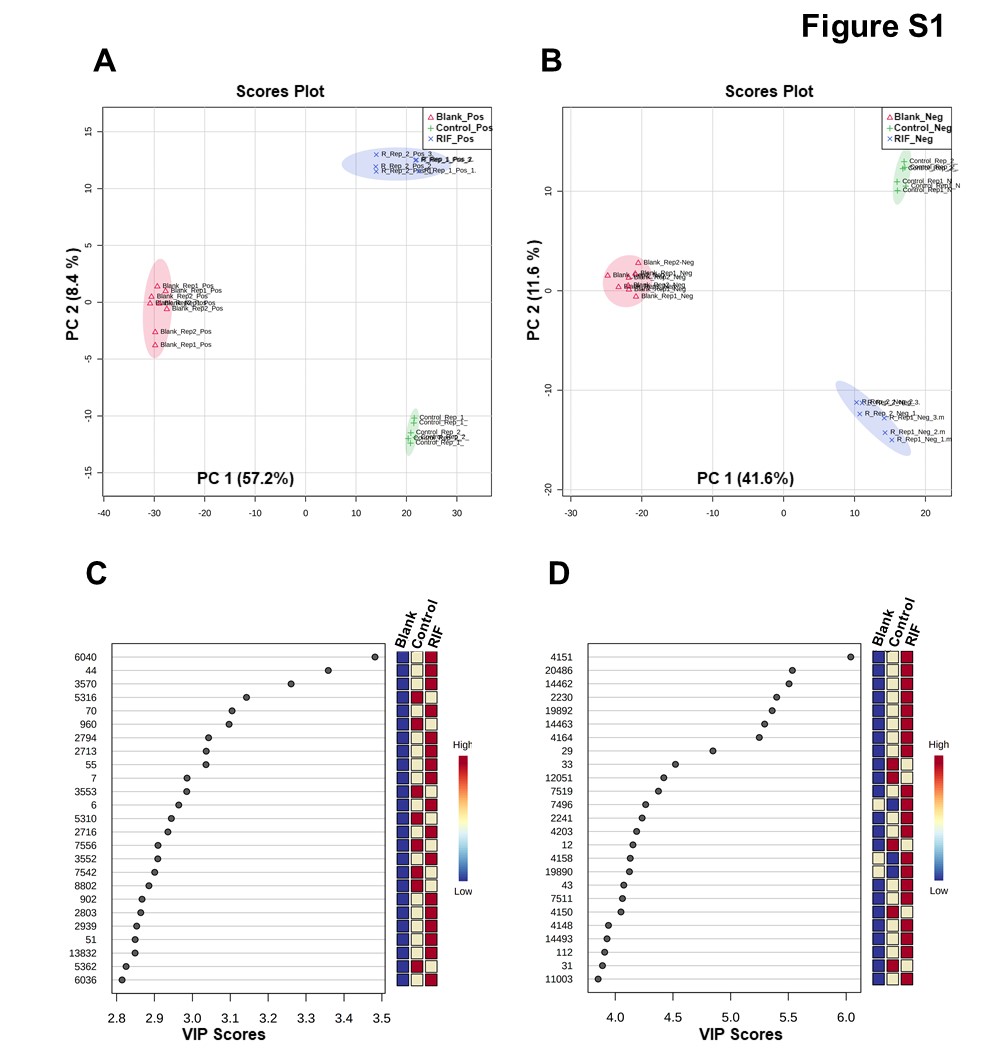

Supplement: Supplementary file 1 [file metabolites-12-00493-s001.zip › Figure S1_140422.jpg]

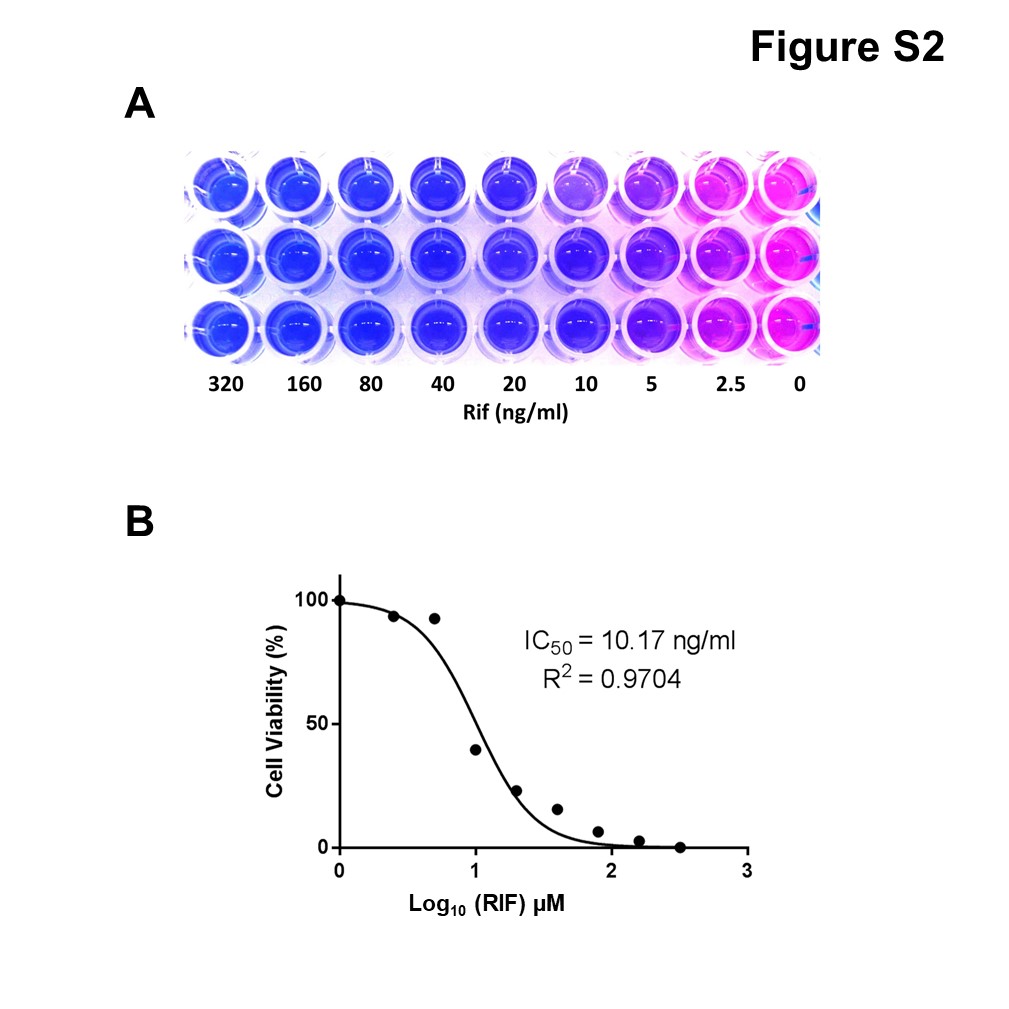

Supplement: Supplementary file 1 [file metabolites-12-00493-s001.zip › Figure S2_140422.jpg]
